# Liquid Biopsies Based on Cell-Free DNA Integrity as a Biomarker for Cancer Diagnosis: A Meta-Analysis

**DOI:** 10.3390/diagnostics14141465

**Published:** 2024-07-09

**Authors:** Ana María Rodríguez-Ces, Óscar Rapado-González, Ángel Salgado-Barreira, María Arminda Santos, Carlos Aroso, Ana Sofia Vinhas, Rafael López-López, María Mercedes Suárez-Cunqueiro

**Affiliations:** 1 Department of Surgery and Medical-Surgical Specialties, Medicine and Dentistry School, Universidade de Santiago de Compostela (USC), 15782 Santiago de Compostela, Spain; anamaria.rodriguez.ces@rai.usc.es (A.M.R.-C.); oscar.rapado@rai.usc.es (Ó.R.-G.); 2 Galician Precision Oncology Research Group (ONCOGAL), Medicine and Dentistry School, Universidade de Santiago de Compostela (USC), 15782 Santiago de Compostela, Spain; rafa.lopez.lopez@gmail.com; 3 Liquid Biopsy Analysis Unit, Translational Medical Oncology Group (ONCOMET), Health Research Institute of Santiago (IDIS), 15706 Santiago de Compostela, Spain; 4 Centro de Investigación Biomédica en Red en Cáncer (CIBERONC), Instituto de Salud Carlos III, 28029 Madrid, Spain; 5 Cancer Biology & Epigenetics Group, Research Center of IPO Porto (CI-IPOP)/CI-IPOP@RISE (Health Research Network), Portuguese Oncology Institute of Porto (IPO Porto)/Porto Comprehensive Cancer Center Raquel Seruca (Porto.CCC), Rua Dr. António Bernardino de Almeida, 4200-072 Porto, Portugal; 6 Department of Public Health, University of Santiago de Compostela, 15782 Santiago de Compostela, Spain; angel.salgado.barreira@usc.es; 7 Consortium for Biomedical Research in Epidemiology and Public Health (CIBER Epidemiology and Public Health—CIBERESP), 28029 Madrid, Spain; 8 Health Research Institute of Santiago de Compostela (IDIS), 15706 Santiago de Compostela, Spain; 9 UNIPRO-Oral Pathology and Rehabilitation Research Unit, University Institute of Health Sciences (IUCS), CESPU, 4585-116 Gandra, Portugal; maria.santos@iucs.cespu.pt (M.A.S.); carlos.ribeiro@iucs.cespu.pt (C.A.); ana.vinhas@iucs.cespu.pt (A.S.V.); 10 Translational Medical Oncology Group (ONCOMET), Health Research Institute of Santiago (IDIS), Complexo Hospitalario Universitario de Santiago de Compostela (CHUS, SERGAS), 15706 Santiago de Compostela, Spain

**Keywords:** cancer, diagnostic accuracy, liquid biopsy, meta-analysis, cell-free DNA

## Abstract

Liquid biopsies have been identified as a viable source of cancer biomarkers. We aim to evaluate the diagnostic accuracy of cell-free DNA integrity (cfDI) in liquid biopsies for cancer. A comprehensive literature search was conducted through PubMed, Embase, Web of Science, and Cochrane Library up to June 2024. Seventy-two study units from forty-six studies, comprising 4286 cancer patients, were identified and evaluated. The Quality Assessment for Studies of Diagnostic Accuracy-2 (QUADAS-2) was used to assess study quality. Meta-regression analysis was employed to investigate the underlying factors contributing to heterogeneity, alongside an evaluation of publication bias. The bivariate random-effect model was utilized to compute the primary diagnostic outcomes and their corresponding 95% confidence intervals (CIs). The pooled sensitivity, specificity, and positive and negative likelihood ratios of cfDI in cancer diagnosis were 0.70 and 0.77, 3.26 and 0.34, respectively. The overall area under the curve was 0.84, with a diagnostic odds ratio of 10.63. This meta-analysis suggested that the cfDI index has a promising potential as a non-invasive and accurate diagnostic tool for cancer. Study registration: The study was registered at PROSPERO (reference No. CRD42021276290).

## 1. Introduction

Cancer keeps its position as one of the predominant causes of death and decreases in life expectancy worldwide [1,2]. According to data from the World Health Organization’s GLOBOCAN network, an estimated 20 million new cases and almost 9.7 million cancer deaths occurred in 2022 [3], and these numbers are not expected to stop growing.

In the era of precision oncology [4,5], molecular biomarkers have been proposed as helpful tools, as they cover a broad range of biochemical entities [6,7], reporting relevant information to improve the diagnosis and decision-making. Although tissue biopsy remains the “gold standard” [8,9], liquid biopsies represent an important approach because they offer the opportunity to detect, analyse, and track cancer in real-time in different body fluids, thus representing a minimally invasive tool [10,11]. Furthermore, they allow us to determine the molecular landscape, including the heterogeneity of the tumor, through different biomarkers during the clinical management of the disease [12,13].

A wide variety of biomarkers exist that can be studied on the different biofluids in the body, each one with different applications and approaches, reporting valuable information not only about the primary tumor and/or metastasis but also the microenvironment, the prognosis of the disease and the response to therapy. These biomarkers include: circulating tumor DNA (ctDNA), that forms a part of the broader category of cell-free DNA (cfDNA), which provides insights into genetic mutations and tumor burden; circulating tumor cells (CTCs), which can indicate the presence of metastasis; tumor-derived extracellular vesicles (tdEVs), which play a role in cell communication and can carry tumor-specific proteins and RNA; circulating tumor RNA (ctRNA), which can reveal gene expression profiles and potential therapeutic targets; and tumor-educated platelets (TEPs), which are involved in the interaction between the tumor and the host’s blood coagulation system and can provide information on tumor growth and metastasis [14].

Among the aforementioned biomarkers, one of the most studied is cfDNA. CfDNA is a mixture of fragmented DNA molecules released from the cells of the body under different physiological and pathological conditions [15,16,17] into different body fluids such as blood, saliva, or urine [18,19,20]. Cancer cells have high anti-apoptotic activity [21], and they release different, arbitrary-size, incompletely digested genomic DNA fragments, resulting from different processes apart from the aforesaid apoptosis, such as necrosis, autophagy, or mitotic catastrophe, into different body fluids [22,23]. On the contrary, healthy cells mostly suffer apoptosis, with an average size of cfDNA ranging between 150–200 bp in plasma [24]. This phenomenon is due to non-random nuclease activity in the inter-nucleosomal linker regions [25], which shows the well-known ‘ladder’ pattern when it is composed of several nucleosomal units [15,26,27,28]. The cell-free DNA integrity (cfDI) index is calculated based on the amplifications by quantitative real-time PCR (qPCR) of a genomic repetitive sequence targeted at the same locus [23,29]. The fragmentation pattern of cfDNA can be characterized using a delta–delta formula based on a threshold cycle (Cp), e^(−ΔΔCp×ln(2))^, or based on a ratio between longer fragments, which are more related to the process of necrosis, to shorter fragments, which are considered to represent the overall amount of cfDNA that stems from the totality of cells [29,30].

The aim of this meta-analysis was to systematically assess and synthesize the results of previously published clinical studies in this field and appraise the overall diagnostic accuracy of the cfDI index across various biofluids for discriminating cancer. We seek to provide a comprehensive assessment of the utility of cfDI as a minimally invasive biomarker for cancer detection, thereby contributing valuable insights that can potentially enhance the precision and effectiveness of cancer diagnostics through liquid biopsy approaches.

## 2. Materials and Methods

### 2.1. Protocol and Registration

The present meta-analysis was conducted in conformity with the Preferred Reporting Items for Systematic Reviews and Meta-analysis (PRISMA) guidelines [31], and the corresponding protocol was registered on PROSPERO International Register of Systematic Reviews (reference No. CRD42021276290).

### 2.2. Search Strategy and Study Selection

We performed a systematic literature search of eligible articles published until 10 June 2024, which was carried out without any language restrictions or specified start dates in several electronic databases, including PubMed, EMBASE, Web of Science, and Cochrane Library. Additionally, free-text searches were performed.

The search strategy was grounded on the following combinations of free-style keywords and Medical Subjects Headings (MeSH): (cell-free DNA OR cfDNA) AND (cancer OR carcinoma OR tumor) AND (integrity index OR DNA integrity OR circulating DNA integrity). All studies were screened through perusal of the titles and abstracts, and manuscripts meeting the inclusion criteria were obtained for comprehensive text evaluation. In addition, we manually scrutinized the reference lists of all original and review articles to identify additional pertinent studies that may have been overlooked in the primary electronic database search.

The literature search for the studies was carried out independently by two investigators (AMRC and ORG), and any disparities were resolved through consensus. Disagreements in study identification during the selection process were reconciled through consensus in conjunction with a third investigator (MMSC).

The studies identified through the search strategy and additional sources were compiled and organized using the RefWorks software (version 2.1.0.1) (https://www.refworks.com/content/path_learn/faqs.asp, accessed on 14 June 2024). The associated tools were utilized to eliminate any duplicate items, and the remaining references were exported to an Excel sheet that contained basic information for screening.

### 2.3. Study Selection

Study inclusion criteria were: (1) liquid biopsies studies based on cfDI as a diagnostic biomarker; (2) studies that enrolled more than 15 patients; (3) inclusion of cancer patients and non-cancer controls (healthy and benign diseases); (4) sufficient data for generating a two-by-two (2 × 2) contingency table containing true-positive (TP), false-positive (FP), true-negative (TN), and false-negative (FN) data; (5) studies that only calculate the cfDI index using the direct cfDNA concentration ratio between a longer fragment to short fragments and/or the delta–delta formula: e^(−ΔΔCp×ln(2))^. The exclusion criteria consisted of the following: (1) reviews, letters, personal opinions, book chapters, case reports, conference abstracts, and meetings; (2) duplicate publications; (3) those experimental in vitro and in vivo studies that reported correlations between liquid biopsies based on cfDI and cancer.

We defined “study unit” (or database) as the analysis of a relationship between cfDI and cancer diagnosis. As different lengths, repetitive DNA sequences chosen as an amplicon, and cfDI calculation methods might be reported in the same manuscript; a single publication may contain multiple study units.

### 2.4. Ethical Statement

All analyses were based on previously published studies and did not involve human beings or experimental subjects. As a result, ethical approval was not necessary.

### 2.5. Data Extraction and Quality Assessment

Data extraction from the eligible full-text articles was carried out independently by two authors (AMRC and ORG) using a structured Microsoft Excel spreadsheet (Microsoft Corp. Redmond, WA, USA). The following information was extracted from each study: author, publication year, journal, country, type of biofluid, type of cancer, number of cases and controls, type of targeted DNA sequence, including length of the amplicons, quantification method, type of cfDI calculation method, and statistical analysis outcomes. If the necessary data were inadequate or required verification, endeavours were made to communicate with the authors to obtain the missing information and minimize reporting bias.

### 2.6. Assessment of Risk Bias

As recommended by the Healthcare Research and Quality Agency, the Quality Assessment of Diagnostic Accuracy Studies-2 checklist (QUADAS-2) was applied by two independent researchers (AMRC and ORG) [32]. Disagreements were resolved by a third reviewer (MMSC) or consensus-based discussion. QUADAS-2 is a tool designed to assess the quality of primary diagnostic accuracy studies, and it consists of 4 principal domains: (1) patient selection, (2) index test, (3) reference standard, and (4) flow of patients and timing of the index test. Each domain was rated for risk of bias and applicability concerns and classified as “low”, “high”, or “unclear”. A score of one was assigned to each item if the response to the risk of bias and applicability questions was “low.” Subsequently, the articles were categorized based on their score into high (6–7 points), moderate (4–5 points), and low (0–3 points) quality groups.

### 2.7. Statistical Analysis

Data analysis was executed using MetaDiSc (version 1.4), R (version 3.4.4), and Stata (version 14.0). For the diagnostic meta-analysis, we determined sensitivity, specificity, positive likelihood ratio (PLR), negative likelihood ratio (NLR), and diagnostic odds ratio (DOR), each accompanied by their 95% confidence intervals (CIs). These calculations were performed using either a bivariate random effects model or a fixed effects model, depending on the context. To evaluate the effectiveness of cfDI in cancer detection, we generated the summary receiver operating characteristic (sROC) curve and computed the area under the curve (AUC). A thorough heterogeneity analysis was conducted to uncover factors impacting accuracy metrics and the choice of statistical model [33]. We utilized Spearman’s correlation and ROC plane plots to investigate heterogeneity caused by the threshold effect, whereas non-threshold heterogeneity was assessed through Cochran’s Q test and I-squared (I2) statistics. Significant heterogeneity was identified by I2 values exceeding 50% and/or *p*-values under 0.05 for Cochran’s Q test. In cases where significant heterogeneity was detected, the DerSimonian and Laird random effects model was applied; otherwise, the Mantel–Haenszel fixed effects model was used. Meta-regression and subgroup analyses were employed to pinpoint potential sources of non-threshold heterogeneity. Furthermore, Fagan’s nomogram was utilized to gauge the predictive value of the cfDI index in diagnosing cancer. Publication bias was evaluated using Deeks’ funnel plot asymmetry test, with statistical significance established at *p* < 0.05.

## 3. Results

### 3.1. Study Selection

**Figure 1:**
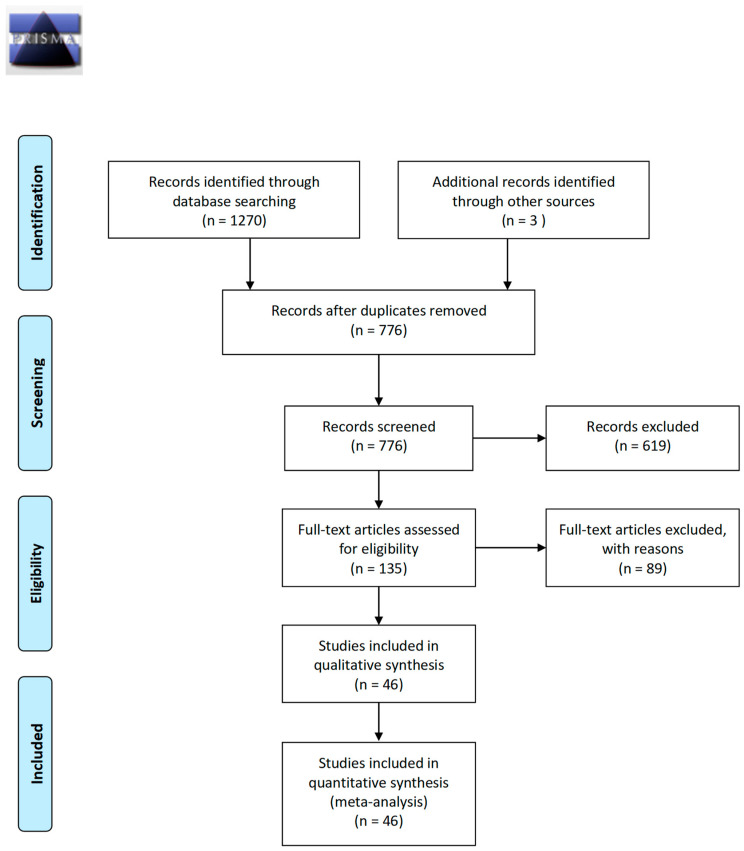
PRISMA flow diagram of literature search and selection criteria. Abbreviations: PRISMA, Preferred Reporting Items for Systematic Reviews and Meta-analysis [31].

As shown in Figure 1, a PRISMA flowchart was used to depict the literature identification and selection process. A total of 1270 eligible published articles were yielded by the electronic database searching, and another three records were identified through a free search and reference checking. After removing duplicates, 776 records were left for the initial title and abstract screening, and 619 were excluded after a review of the text. Finally, 135 articles that are closely related to the cfDI index as a diagnostic tool for cancer were selected for full-text screening and further assessment of their eligibility. A review of the full-text articles led to the exclusion of 89 and the inclusion of 46 articles as part of our present meta-analysis [29,30,34,35,36,37,38,39,40,41,42,43,44,45,46,47,48,49,50,51,52,53,54,55,56,57,58,59,60,61,62,63,64,65,66,67,68,69,70,71,72,73,74,75,76,77].

### 3.2. Characteristics of Included Studies

The main characteristics of the included studies are presented in Table 1. The selection process yielded a total of 46 studies, which, in turn, encompassed 72 study units. These papers focus on different types of cancer, from the most prevalent, such as breast, lung, and prostate cancer, to others with a lower incidence, such as ampullary carcinoma cancer, in which cfDI can be used as a diagnostic tool. In total, 4286 cases (sample sizes ranging from 16 to 148) and 3270 controls (ranging from 10 to 132) were included. All articles were published between 2003 and 2024 and were conducted in the following geographical regions: China (14), Egypt (12), Germany (7), EEUU (4), India (2), Italy (1), Poland (1), Korea (1), Australia (1), Canada (1), Spain (1), and Lithuania (1). In all cases, cfDI was detected by qPCR. Regarding the fluids used, plasma, serum, and other biofluids (pleural effusions, saliva, and urine) were the biofluids chosen for the cfDNA extraction. Regarding the methods used to calculate the cfDI index, a simple ratio of the concentrations of longer DNA fragments to shorter fragments of a targeted DNA sequence was used in 60 study units. On the other side, the drawn-up formula based on Cp value differences described by Wang [29] was used in only 12 study units. *Arthrobacter luteus* (ALU) was the most used targeted repetitive sequence for calculating the ratio between lengths, chosen in 65.28% of the study units (47/72). β-actin (ACTB) was in second place (13/72), and the other proposed repetitive elements included LINE-1 (Long Interspersed Nuclear Elements) (5/72), GAPDH (Glyceraldehyde-3-Phosphate Dehydrogenase) (4/72), ERV (Endogenous Retrovirus) gene (2/52), and APP gene (Amyloid Precursor Protein) (1/72) that were chosen with less frequency. The length of the DNA sequences used for ratio calculation had a wide variability between studies, the most popular being the one that divides 247 to 115 base pairs of ALU repetitive sequences (35/72).

### 3.3. Quality Assessment of the Included Studies

The studies included in this meta-analysis showed an average QUADAS-2 score of 5.76. In terms of the risk of bias, numerous studies did not specify patient selection (random or consecutive) and did not provide a detailed description of the inclusion/exclusion criteria. Regarding the index test, most studies report threshold values for classifying the disease through the cfDI index, which could overestimate its predictive value. Additionally, regarding flow and timing, various studies were not clear about the appropriate intervals between the index test and reference test. The reference standard was specified clearly in the majority of studies. In terms of applicability, all domains showed a low risk of bias. An overview of the quality characteristics of included studies with QUADAS-2 template is shown in Supplementary Table S1, Supplemental Digital Content.

### 3.4. Diagnostic Accuracy of Cell-Free DNA Integrity Index

**Figure 2:**
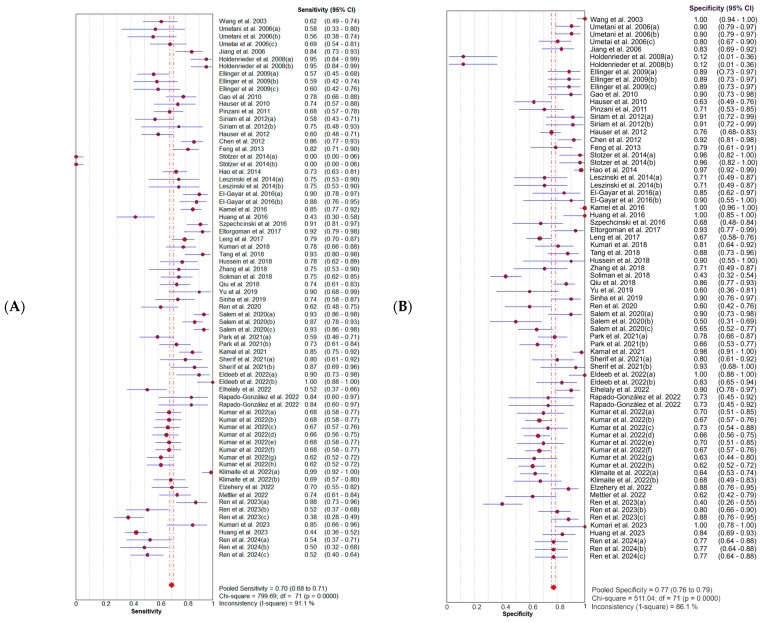
Forest plot of (**A**) sensitivities and (**B**) specificities from test-accuracy studies of cfDI in liquid biopsies for predicting cancer diagnosis [29,30,34,35,36,37,38,39,40,41,42,43,44,45,46,47,48,49,50,51,52,53,54,55,56,57,58,59,60,61,62,63,64,65,66,67,68,69,70,71,72,73,74,75,76,77]. Abbreviations: cfDI = cell-free DNA integrity.

**Figure 3:**
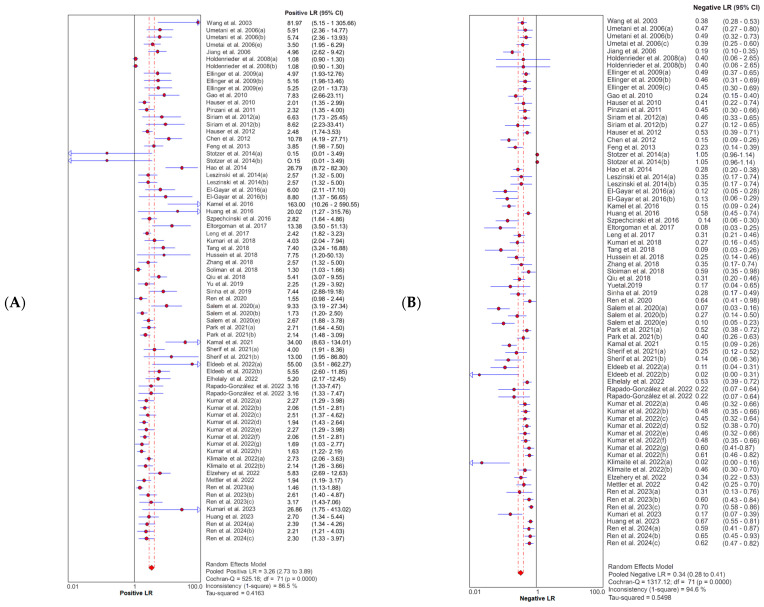
Forest plot of likelihood ratios for (**A**) positive and (**B**) negative test results from cfDI in liquid biopsies studies for predicting cancer [29,30,34,35,36,37,38,39,40,41,42,43,44,45,46,47,48,49,50,51,52,53,54,55,56,57,58,59,60,61,62,63,64,65,66,67,68,69,70,71,72,73,74,75,76,77]. Abbreviations: cfDI = cell-free DNA integrity.

**Figure 4:**
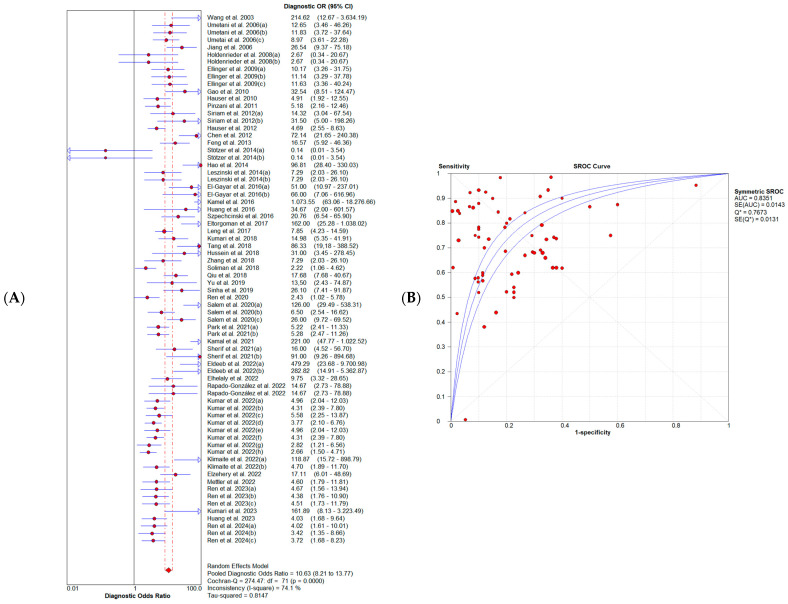
Pooled performance of cfDI based on liquid biopsies in cancer diagnosis [29,30,34,35,36,37,38,39,40,41,42,43,44,45,46,47,48,49,50,51,52,53,54,55,56,57,58,59,60,61,62,63,64,65,66,67,68,69,70,71,72,73,74,75,76,77]. (**A**) Forest plot displaying the pooled DOR of cfDI. (**B**) sROC curve illustrating the pooled estimates of sensitivity, specificity, and the AUC of cfDI. Q* is the point on the sROC curve where the sensitivity equals the specificity. Abbreviations: AUC = area under the curve; cfDI = cell-free DNA integrity; DOR = diagnostic odds ratio; sROC = summary receiver operator characteristic.

SE and 95% CIs for sensitivity and specificity estimates of a total of 72 study units were included in this meta-analysis. The forest plots (Figure 2) illustrate that the combined diagnostic sensitivity and specificity of the cfDI index were 0.70 (95% CI: 0.68–0.71) and 0.77 (95% CI: 0.76–0.79), respectively. This corresponds to a pooled positive likelihood ratio (PLR) of 3.26 (95% CI: 2.73–3.89) and a negative likelihood (NLR) of 0.34 (95% CI: 0.28–0.41) (Figure 3). The summary DOR was 10.63 (95% CI: 8.21–13.77), and the AUC was 0.84 (95% CI: 0.81–0.86) with a standard error (SE) of 0.014, indicating the overall diagnostic performance of the cfDI index (Figure 4). The Q-value for the sROC curve was 0.77 (95% CI: 0.74–0.79) with an SE of 0.01.

Fagan’s nomogram plot was generated for visually presenting diagnostic performance (see Figure S2, Supplemental Digital Content); given a pre-test probability of 43%, a positive measurement leads to a post-test cancer probability of 71%, whereas a negative measurement leads to a post-test probability of 20%.

As shown in Figure 2, Figure 3 and Figure 4, the I2 of sensitivity, specificity, PLR, NLR, and DOR were 91.1% (*p* < 0.0001), 86.1% (*p* < 0.0001), 86.5% (*p* < 0.0001), 94.6% (*p* < 0.0001), and 74.1% (*p* < 0.0001), respectively. These results of the diagnostic meta-analysis demonstrated that a substantial heterogeneity exists between the included studies. Furthermore, a typical pattern of the “shoulder arm” plot shown in the ROC space, which contains the representation of accuracy, suggests the presence of a threshold effect (see Figure S3, Supplemental Digital Content).

The Spearman rank correlation coefficient between the logit of sensitivity and the logit of 1-specificity was determined to be 0.159 (*p* = 0.183). This suggests that, in addition to the threshold effect, there may be other factors contributing to variations in accuracy estimates among individual studies.

In addition to the threshold effect test, a meta-regression analysis was carried out to explore the possible sources of heterogeneity using the following covariates as predictor variables: the biofluids tested (serum, plasma, and others), the targeted gen used to the integrity index formula (ALU vs. others), the type of controls (healthy, benign, or non-cancer controls), and the cohort size (*n* ≥ 100 vs. *n* < 100). None of these variables reported any kind of effect on the diagnostic accuracy of cancer (see Table S1, Supplemental Digital Content). Subsequently, subgroup analysis was performed to further explore the potential diagnostic value of cfDI in liquid biopsies. No statistically significant differences were observed in the subgroup analysis, suggesting that none of the subgroups report a potential resource heterogeneity. The results of the subgroup analysis are shown in Table 2.

### 3.5. Publication Bias

The assessment of potential publication bias in each of the selected studies was conducted using Deek’s funnel plot asymmetry test, which yielded an overall slope coefficient *p*-value greater than 0.05. The observed data pattern exhibits symmetry, indicating no evidence of publication bias in the pooled analysis. Therefore, our meta-analysis suggests the absence of significant publication bias (refer to Figure S4, Supplemental Digital Content).

## 4. Discussion

Although the 5-year relative survival rate has improved in the past decades for most cancer types [78], it is still necessary to keep developing early diagnostic strategies in order to increase the quality of life the chances of successful treatment for patients [79], and reduce the healthcare costs. In the last few years, liquid biopsies have emerged as an attractive source of molecular biomarkers for cancer diagnosis. Specifically, cfDNA analysis has shown potential as a tool for diagnosis and cancer management [80,81,82]. It was described in blood for the first time in 1948 [83], but it was not until 1977 that it started to be studied for its possible link with cancer regarding cfDNA quantification [84]. However, the cfDNA concentration is not cancer-specific, as elevated levels of cfDNA have been detected as well in other physiological and pathological conditions [85,86,87,88]. Apart from the cfDNA concentration, cfDI has been proposed as a diagnostic cancer biomarker. Several studies have observed that the cfDNA fragmentation pattern in cancer patients is different from that in non-cancer individuals [89,90,91]. A great number of fragments longer than 180 bp have been detected in cancer, which leads to the possibility of a necrotic cell death origin [25,92]. Based on this theory, the cfDI index was used in numerous studies as a potential cancer biomarker [49,55,62]. Initially, a formula based on the threshold (Cp) value for each qPCR reaction was proposed [29]. Subsequently, a method was developed to measure the integrity of circulating DNA using ALU repeats, which are the most abundant repetitive elements in the genome, with a simple ratio between the longer and the shorter DNA sequences [34]. Although these two methods are different from each other, they report similar measurements [45,47], and both set the stage for the following researchers who keep using repeated sequences of the human genome to measure the integrity index.

To the best of our knowledge, this article is the first meta-analysis to evaluate the diagnostic accuracy of cfDI in different liquid biopsies in cancer patients. A total of 46 articles (72 study units) involving 4286 cases and 3270 controls have been analyzed. According to QUADAS-2 quality evaluation, most of the included studies showed moderate quality. To incorporate cfDI as an early, cost-effective cancer diagnostic proof by itself in clinical settings, the specificity and sensitivity of the test very close to 100% should be achieved. In the present study, the overall diagnostic accuracy of the cfDI for discriminating between cancer and non-cancer patients reported pooled sensitivity and specificity values of 0.70 and 0.77, respectively. These results indicate a moderate sensitivity and a moderate–high diagnostic specificity. Furthermore, no evidence of publication bias among eligible studies was observed, indicating that the results of the present meta-analysis are reliable.

The summary DOR of 10.63 (95% CI: 8.21–13.77) reflects the diagnostic capacity of cfDI for cancer without being influenced by its prevalence among the population. This value indicates that the odds of having a high cfDI level for cancer patients are more than 15 times higher than the odds for the controls. For its part, the AUC under sROC for cfDI was 0.84, showing its diagnostic capacity. The pooled PLR value of 3.26 means that a person with a high cfDI index has a little more than a 3-fold higher probability of having cancer than a control. On the contrary, the pooled NLR results indicated that a person with a low cfDI index had only a 34% probability of not having cancer.

Additionally, given a pre-test probability of 43% in Fagan’s nomogram, correct cancer diagnosis increased to 71% after a positive test (high values of the cfDI index) and reduced to 34% after a negative test (low value of the cfDI index). Overall, all the present results suggest that cfDI is quite specific, which might be helpful in the assessment of cancer diagnosis, but it shows relative sensitivity as a cancer diagnostic biomarker. As cancer involves a complex cascade of molecular events, some studies have combined the cfDI index with other biomarkers, showing an improvement in diagnostic accuracy [36,46,55,58,60]. However, this meta-analysis was not evaluated because there was not enough data to carry out an analysis.

Heterogeneity is a significant factor in any diagnostic accuracy meta-analysis; therefore, the possible reasons that could lead to inconsistencies across the studies should be evaluated. In the present work, a high heterogeneity was observed, so a bivariate random-effect model was applied. Despite the fact that the threshold effect is one of the primary causes of heterogeneity in diagnostic test accuracy studies [33], we could not demonstrate with the ROC plane or Spearman correlation the visual deviation denoted by accuracy estimates on forest plots. Furthermore, other possible heterogeneity sources were analyzed, but no significant differences were found among the subgroups. However, other reasons might explain this great heterogeneity in accuracy estimates, such as sociodemographic characteristics (e.g., gender, ethnicity, age, or lifestyle) or clinicopathological conditions (e.g., TNM staging, tumor anatomic location, histological type, or other concomitant diseases). Nonetheless, there was not a sufficient number of articles to assess these variables as a group by themselves.

Apart from the heterogeneity drawback already explained and the different biological effects that were not considered in most of the included studies, other limitations were presented in this meta-analysis. First, the majority of the studies used the DNA repetitive elements’ length reported in blood to evaluate cfDI in other liquid biopsies. However, for example, DNase I enzymatic activity is more than 100-fold higher in urine than in the blood [93,94], which leads to smaller size DNA fragments (around 82 bp long) [95,96]. In this sense, future investigations should be carried out to establish the proper ratio for each body effluent [97]. Last, because of the different thresholds set in the included studies to define a positive test result, a lack of standardization is denoted, which is one of the reasons for the differences seen in the sensitivities and specificities of the test accuracy of the studies. In this line, it is necessary to standardize both the pre-analytical and analytical steps in data interpretation, including cut-off determination.

## 5. Conclusions and Future Perspectives

From a clinical perspective, cfDI shows promising value as a minimally invasive biomarker for cancer management. The present research provides updated evidence on the diagnostic potential of cfDI across a wide variety of malignancies, highlighting and reinforcing the growing interest in cfDI as a tumor biomarker. Additionally, this work details the design and methodological characteristics of each included study, emphasizing the significant variability among different research efforts that could influence the overall diagnostic performance of this biomarker. In this sense, subgroup analyses based on the type of biofluid, sample size, DNA repetitive sequence, and type of control were carried out to understand the heterogeneity among the studies better; however, no significant differences in terms of diagnostic accuracy were observed, which suggests the importance to evaluate the impact of other factors in the performance of this biomarker. Therefore, future perspectives must focus on developing robust studies using standardized protocols to establish the real clinical value of cfDI as a liquid biopsy diagnostic tool. For that, it is important to better understand the biological significance of cfDI due to the behavior of this biomarker varying in the different liquid biopsies and malignancies. In this line, the advances in molecular biology techniques, such as next-generation sequencing platforms, could contribute to providing more information about the size of cfDNA fragments and cfDI under different physiological and pathological conditions. Moreover, the cfDI index could be combined with other cancer-related biomarkers, such as conventional tumor markers or epigenetic alterations, improving the performance of liquid biopsies for cancer detection.

Overall, our investigation provides updated knowledge and valuable insights into various aspects of cfDI as a cancer biomarker, identifying the main limitations of the research in this field, which will help the medical and scientific community to address future well-design studies for advancing in the implementation of liquid biopsy tools for cancer diagnosis.

## Figures and Tables

**Table 1:** Summary of descriptive characteristics of included studies (*n* =46).

First Author	Country	Type of Biofluid	*N* Case	Type of Cancer	*N* Control	Type of Control	Repetitive Sequence	Ratio	Method
Wang et al. (2003) [29]	EE.UU.	Plasma	61	Gynecological and BC	65	Benign	β-actin (ACTB)	400/100	qPCR
Umetani et al. (2006) [34]	EE.UU.	Serum	19	PACs	51	Healthy	ALU	275/115	qPCR
		Serum	32	CRC	51	Healthy	ALU	275/115	qPCR
Umetai et al. (2006) [30]	EE.UU.	Serum	51	BC	51	Healthy	ALU	247/115	qPCR
Jiang et al. (2006) [35]	EE.UU.	Plasma	58	HNSCC	47	Healthy	β-actin (ACTB)	400/100	qPCR
Holdenrieder et al. (2008) [36]	Germany	Serum	42	Cancer *	17	Benign	ERV gene	347/137	qPCR
		Plasma	42		17	Benign	ERV gene	347/137	qPCR
Ellinger et al. (2009 [37])	Germany	Serum	74	Testicular	35	Healthy	β-actin (ACTB)	384/106	qPCR
		Serum	39		35	Healthy	β-actin (ACTB)	384/106	qPCR
		Serum	35		35	Healthy	β-actin (ACTB)	384/106	qPCR
Gao et al. (2010) [38]	China	Plasma	60	Leukemia	30	Healthy	β-actin (ACTB)	384/106	qPCR
Hauser et al. (2010) [39]	Germany	Serum	35	RCC	54	Healthy	β-actin (ACTB)	384/106	qPCR
Pinzani et al. (2011) [40]	Italy	Plasma	79	Melanoma	34	Healthy	APP gene	180/67	qPCR
Sriram et al. (2012) [41]	Australia	Pleural Fluid	52	MPES	23	Benign	ALU	247/115	qPCR
		Pleural Fluid	16	Mesothelioma	23	Benign	ALU	247/115	qPCR
Hauser et al. (2012 [42])	Germany	Serum	75	BCA	132	Non-Cancer Control ***	β-actin (ACTB)	384/106	qPCR
Chen et al. (2012 [43])	China	Serum	80	HCC	50	Healthy	β-actin (ACTB)	400/100	qPCR
Feng et al. (2013) [44]	China	Plasma	71	PC	33	Benign	ALU	247/115	qPCR
Stötzer et al. (2014) [45]	Germany	Plasma	65	BC	28	Healthy	ALU	247/115	qPCR
		Plasma	65		28	Healthy	ALU	247/115	qPCR
Hao et al. (2014) [46]	China	Serum	104	CRC	110	Healhty	ALU	247/115	qPCR
Leszinski et al. (2014) [47]	Germany	Serum	24	CRC	24	Healthy	ALU	247/115	qPCR
		Serum	24		24	Healthy	ALU	247/115	qPCR
El-Gayar et al. (2016) [48]	Egypt	Serum	50	CRC	20	Healthy	ALU	247/115	qPCR
		Serum	50		10	Benign	ALU	247/115	qPCR
Kamel et al. (2016) [49]	Egypt	Plasma	95	BC	95	Benign	β-actin (ACTB)	400/100	qPCR
Huang et al. (2016) [50]	China	Plasma	53	HCC	22	Healthy	ALU	247/115	qPCR
Szpechcinski et al. (2016) [51]	Polonia	Plasma	65	NSCLC	28	Benign	β-actin (ACTB)	400/100	qPCR
Eltorgoman et al. (2017) [52]	Egypt	Pleural Effusions	39	Malignant Effusions **	29	Benign	ALU	247/115	qPCR
Leng et al. (2017) [53]	China	Plasma	106	NSCLC	107	Healthy	ALU	247/115	qPCR
Kumari et al. (2018) [54]	India	Serum	60	GBC	36	Non-Cancer Control	ALU	247/115	qPCR
Tang et al. (2018) [55]	China	Serum	40	BC	40	Healthy	ALU	247/115	qPCR
Hussein et al. (2018) [56]	Egypt	Plasma	40	BC	10	Healthy	ALU	247/115	qPCR
Zhang et al. (2018) [57]	China	Plasma	24	OC	24	Non-Cancer Control	ALU	219/115	qPCR
Soliman et al. (2018) [58]	Egypt	Serum	60	LC	80	Non-Cancer Control	ALU	247/115	qPCR
Qiu et al. (2018) [59]	China	Serum	68	EC	81	Non-Cancer Control	ALU	247/115	qPCR
Yu et al. (2019) [60]	China	Plasma	20	OC	20	Healthy	ALU	260/111	qPCR
Sinha et al. (2019) [61]	Canada	Plasma	39	CRC	40	Healthy	ALU	265/80	qPCR
Ren et al. (2020) [62]	China	Urine	55	NSCLC	35	Healthy	LINE-1	266/97	qPCR
Salem et al. (2020) [63]	Egypt	Serum	90	CRC	30	Healthy	ALU	247/115	qPCR
		Serum	90		30	Benign	ALU	247/115	qPCR
		Serum	90		60	Non-Cancer Control	ALU	247/115	qPCR
Park et al. (2021) [64]	Korea	Plasma	64	BC	64	Healthy	ALU	263/58	qPCR
		Plasma	64	LC	64	Healthy	ALU	263/58	qPCR
Kamal et al. (2021) [65]	Egypt	Plasma	80	HCC	80	Benign	ALU	247/115	qPCR
Sherif et al. (2021) [66]	Egypt	Plasma	30	EOC	30	Benign	ALU	247/115	qPCR
		Plasma	30		15	Healthy	ALU	247/115	qPCR
Eldeeb et al. (2022 [67])	Egypt	Plasma	30	HCC	30	Healthy	ALU	247/115	qPCR
		Plasma	30		30	Benign	ALU	247/115	qPCR
Elhelaly et al. (2022) [68]	Egypt	Serum	50	BC	50	Benign	ALU	247/115	qPCR
Rapado-González et al. (2022) [69]	Spain	Saliva	19	OSCC	15	Healthy	ALU	274/60	qPCR
		Saliva	19		15	Healthy	ALU	115/60	qPCR
Kumar et al. (2022) [70]	Egypt	Serum	100	HCC	30	Healthy	ALU	247/115	qPCR
		Serum	100		100	Non-Cancer Control	ALU	247/115	qPCR
		Serum	100		30	Healthy	GAPDH	205/110	qPCR
		Serum	100		100	Non-Cancer Control	GAPDH	205/110	qPCR
		Serum	100		30	Healthy	ALU	247/115	qPCR
		Serum	100		100	Non-Cancer Control	ALU	247/115	qPCR
		Serum	100		30	Healthy	GAPDH	205/110	qPCR
		Serum	100		100	Non-Cancer Control	GAPDH	205/110	qPCR
Klimaite et al. (2022) [71]	Lithuania	Plasma	68	PTC	86	Healthy	β-actin (ACTB)	394/99	qPCR
		Plasma	68	PTC	31	Benign	β-actin (ACTB)	394/99	qPCR
Elzehery et al. (2022) [72]	Egypt	Serum	50	HCC	50	Non-Cancer Control	ALU	247/115	qPCR
Mettler et al. (2022) [73]	Germany	Plasma	62	NEN	29	Healthy	LINE-1	266/97	qPCR
Ren et al. (2023) [74]	China	Plasma	40	NSCLC	50	Healthy	ALU	115/60	qPCR
			44		50	Healthy	ALU	115/60	qPCR
			84		50	Healthy	ALU	115/60	qPCR
Kumari et al. (2023) [75]	India	Serum	27	OPSCC	15	Healthy	ALU	247/115	qPCR
Huang et al. (2023) [76]	China	Plasma	148	LSCC	43	Non-Tumor Control	ALU	247/115	qPCR
Ren et al. (2024) [77]	China	Plasma	37	NSCLC	53	Healthy	LINE-1	266/97	qPCR
			34		53	Healthy	LINE-1	266/97	qPCR
			71		53	Healthy	LINE-1	266/97	qPCR

* Cancer patients included neoplastic diseases gastrointestinal (gastric, colonic, rectal, and pancreatic cancer; *n* = 18), gynecologic (ovarian, cervical, endometrial, and breast cancer; *n* = 14), and urologic (prostate, renal, and bladder cancer; *n* = 10). ** Malignant pleural effusions included malignant pleural mesothelioma (*n* = 5), metastatic cancer originating from adenocarcinoma of the lung, breast cancer, and lymphoma (*n* = 34). *** Non-cancer control group included studies that consider benign patients and healthy individuals without any distinction as the control group. Abbreviations: BC = breast cancer; CRC = colorectal cancer; GBC = gallbladder cancer; HCC = hepatocellular carcinoma; OC = ovarian cancer; PACs = periampullary adenocarcinomas; NSCLC = non-small-cell lung carcinoma; RCC = renal cell carcinoma; MPEs = malignant pleural effusions, BCA = bladder cancer; HCC = hepatocellular carcinoma; LC = lung cancer; PC = prostate cancer; EC = esophageal carcinoma; EOC = epithelial ovarian cancer; OSCC = oral squamous cell carcinoma; PTC = Papillary thyroid carcinoma; NEN = Neuroendocrine neoplasms; OPSCC = oropharyngeal squamous cell carcinoma; LSCC = laryngeal squamous cell carcinoma; ALU = *Arthrobacter luteus*; ACTB = β-actin, APP gene = Amyloid Precursor Protein, ERV = Endogenous Retrovirus, LINE = Long Interspersed Nuclear Element, GAPDH = Glyceraldehyde-3-Phosphate Dehydrogenase; qPCR = quantitative real-time PCR.

**Table 2:** Subgroup analysis of cfDI index for cancer diagnosis based on different covariates.

Subgroups	No of Study Units	Sensitivity (95% CI)	I^2^ (%)	Specificity (95% CI)	I^2^ (%)	PLR (95% CI)	I^2^ (%)	NLR (95% CI)	I^2^ (%)	DOR (95% CI)	I^2^ (%)	AUC (95% CI)
**Biofluid**												
Plasma	33	0.66 (0.64–0.68)	94.6	0.79 (0.77–0.81)	87.8	3.21 (2.4–4.31)	87.5	0.34 (0.24–0.47)	97.2	10.88 (7.15–16.55)	73.5	0.84 (0.8–0.89)
Serum	33	0.73 (0.71–0.75)	81	0.75 (0.73–0.77)	85.8	3.26 (2.53–4.13)	87.2	0.36 (0.31–0.42)	72.5	9.99 (7.05–14.16)	75.9	0.82 (0.78–0.86)
Others *	6	0.72 (0.65–0.78)	76.1	0.8 (0.72–0.86)	69.6	4.17 (1.96–8.87)	74.3	0.30 (0.17–0.53)	75	16.37 (4.64–57.81)	76.1	0.87 (0.76–0.98)
**DNA repetitive sequence**												
ALU	47	0.69 (0.67–0.71)	92.7	0.79 (0.77–0.81)	84.2	3.63 (2.95–4.48)	79.5	0.31 (0.24–0.40)	96.4	12.82 (9.24–17.80)	72.4	0.86 (0.83–0.89)
Others **	25	0.71 (0.69–0.73)	85.6	0.75 (0.72–0.77)	88.7	2.67 (1.99–3.59)	90.6	0.41 (0.34–0.49)	76	7.64 (5.09–11.46)	74.7	0.80 (0.75–0.85)
**N size**												
<100	39	0.69 (0.67–0.71)	92.6	0.78 (0.76–0.80)	82.5	3.44 (2.52–4.68)	89.2%	0.31 (0.23–0.43)	96.4	11.62 (7.99–16.89)	63	0.84 (0.81–0.88)
>100	33	0.70 (0.68–0.72)	88.8	0.77 (0.75–0.79)	89.1	3.07 (2.5–3.76)	81.9	0.37 (0.31–0.44)	85.2	9.81 (6.87–14.02)	80.8	0.82 (0.78–0.87)
**Control**												
Healthy	44	0.67 (0.65–0.69)	92.3	0.79 (0.77–0.81)	79.6	3.2 (2.66–3.86)	70.9	0.35 (0.27–0.45)	96	10.20 (7.36–14.15)	70.7	0.84 (0.81–0.88)
Benign	16	0.81 (0.78–0.83)	84.4	0.84 (0.81–0.87)	92.2	5.25 (2.51–10.97)	96.7	0.25 (0.18–0.35)	78.5	24.02 (11.50–50.14)	73.5	0.90 (0.86–0.94)
Non-Cancer Control ***	12	0.67 (0.64–0.70)	86.3	0.70 (0.67–0.73)	81.9	2.40 (1.9–3.05)	76.4	0.43 (0.35–0.54)	76	6.06 (3.98–9.22)	73	0.77 (0.72–0.83)

* Other biofluids included pleural effusions, pleural fluid, urine, and saliva. ** Other DNA repetitive sequences included β-actin (ACTB), APP gene (Amyloid Precursor Protein), ERV gene (Endogenous Retrovirus), and LINE-1 (long interspersed nuclear elements), GAPDH = Glyceraldehyde-3-Phosphate Dehydrogenase. *** Non-cancer control group included studies that consider benign patients and healthy individuals without any distinction as the control group. Abbreviations: ALU = *Arthrobacter luteus*; AUC = area under the curve; DOR = diagnostic odds ratio; PLR = positive likelihood ratio; NLR = negative likelihood ratio; CI = confidence interval.

## Data Availability

All data generated or analyzed during this study are included in this published article.

## Supplementary Materials

The following supporting information can be downloaded at: https://www.mdpi.com/article/10.3390/diagnostics14141465/s1, Figure S1: Quality assessment of the included studies according to Quality Assessment of Diagnostic Accuracy Studies-2 (QUADAS-2) template. Figure S2: Fagan’s monogram evaluating the clinical utility of liquid biopsies cfDI for differentiating cancer patients. Figure S3: SROC space for the assessment of the threshold effect of cfDI in liquid biopsies on cancer. Figure S4: Deeks’ funnel plot asymmetry test for the assessment of potential bias of included studies. Table S1. Results of regression meta-analysis.

## References

[1] Naghavi M., Ong K.L., Aali A., Ababneh H.S., Abate Y.H., Abbafati C., Abbasgholizadeh R., Abbasian M., Abbasi-Kangevari M., Abbastanbar H. (2024). GBD 2021 Causes of Death Collaborators. Global Burden of 288 Causes of Death and Life Expectancy Decomposition in 204 Countries and Territories and 811 Subnational Locations, 1990–2021: A Systematic Analysis for the Global Burden of Disease Study 2021. Lancet.

[2] Dunn J. (2023). It Is Time to Close the Gap in Cancer Care. JCO Glob. Oncol..

[3] Bray F., Laversanne M., Sung H., Ferlay J., Siegel R.L., Soerjomataram I., Jemal A. (2024). Global Cancer Statistics 2022: GLOBOCAN Estimates of Incidence and Mortality Worldwide for 36 Cancers in 185 Countries. CA Cancer J. Clin..

[4] König I.R., Fuchs O., Hansen G., von Mutius E., Kopp M.V. (2017). What Is Precision Medicine?. Eur. Respir. J..

[5] Malone E.R., Oliva M., Sabatini P.J.B., Stockley T.L., Siu L.L. (2020). Molecular Profiling for Precision Cancer Therapies. Genome Med..

[6] Ashley E.A. (2016). Towards Precision Medicine. Nat. Rev. Genet..

[7] Das S., Dey M.K., Devireddy R., Gartia M.R. (2023). Biomarkers in Cancer Detection, Diagnosis, and Prognosis. Sensors.

[8] Chen M., Zhao H. (2019). Next-Generation Sequencing in Liquid Biopsy: Cancer Screening and Early Detection. Hum. Genom..

[9] Tay T.K.Y., Tan P.H. (2021). Liquid Biopsy in Breast Cancer: A Focused Review. Arch. Pathol. Lab. Med..

[10] Poulet G., Massias J., Taly V. (2019). Liquid Biopsy: General Concepts. Acta Cytol..

[11] Im Y.R., Tsui D.W.Y., Diaz L.A., Wan J.C.M. (2021). Next-Generation Liquid Biopsies: Embracing Data Science in Oncology. Trends Cancer.

[12] Shegekar T., Vodithala S., Juganavar A. (2023). The Emerging Role of Liquid Biopsies in Revolutionising Cancer Diagnosis and Therapy. Cureus.

[13] Sisodiya S., Kasherwal V., Khan A., Roy B., Goel A., Kumar S., Arif N., Tanwar P., Hussain S. (2023). Liquid Biopsies: Emerging Role and Clinical Applications in Solid Tumours. Transl. Oncol..

[14] Wu J., Hu S., Zhang L., Xin J., Sun C., Wang L., Ding K., Wang B. (2020). Tumor Circulome in the Liquid Biopsies for Cancer Diagnosis and Prognosis. Theranostics.

[15] Lo Y.M.D., Han D.S.C., Jiang P., Chiu R.W.K. (2021). Epigenetics, Fragmentomics, and Topology of Cell-Free DNA in Liquid Biopsies. Science.

[16] Heitzer E., Haque I.S., Roberts C.E.S., Speicher M.R. (2019). Current and Future Perspectives of Liquid Biopsies in Genomics-Driven Oncology. Nat. Rev. Genet..

[17] Qi T., Pan M., Shi H., Wang L., Bai Y., Ge Q. (2023). Cell-Free DNA Fragmentomics: The Novel Promising Biomarker. Int. J. Mol. Sci..

[18] Lousada-Fernandez F., Rapado-Gonzalez O., Lopez-Cedrun J.-L., Lopez-Lopez R., Muinelo-Romay L., Suarez-Cunqueiro M.M. (2018). Liquid Biopsy in Oral Cancer. Int. J. Mol. Sci..

[19] Rapado-González Ó., Rodríguez-Ces A.M., López-López R., Suárez-Cunqueiro M.M. (2023). Liquid Biopsies Based on Cell-Free DNA as a Potential Biomarker in Head and Neck Cancer. Jpn. Dent. Sci. Rev..

[20] Chauhan P.S., Shiang A., Alahi I., Sundby R.T., Feng W., Gungoren B., Nawaf C., Chen K., Babbra R.K., Harris P.K. (2023). Urine Cell-Free DNA Multi-Omics to Detect MRD and Predict Survival in Bladder Cancer Patients. NPJ Precis. Oncol..

[21] de Bruin E.C., Medema J.P. (2008). Apoptosis and Non-Apoptotic Deaths in Cancer Development and Treatment Response. Cancer Treat. Rev..

[22] Bronkhorst A.J., Ungerer V., Holdenrieder S. (2019). The Emerging Role of Cell-Free DNA as a Molecular Marker for Cancer Management. Biomol. Detect. Quantif..

[23] Kustanovich A., Schwartz R., Peretz T., Grinshpun A. (2019). Life and Death of Circulating Cell-Free DNA. Cancer Biol. Ther..

[24] Corcoran R.B., Chabner B.A. (2018). Application of Cell-Free DNA Analysis to Cancer Treatment. N. Engl. J. Med..

[25] Thierry A.R. (2023). Circulating DNA Fragmentomics and Cancer Screening. Cell Genom..

[26] El Messaoudi S., Rolet F., Mouliere F., Thierry A.R. (2013). Circulating Cell Free DNA: Preanalytical Considerations. Clin. Chim. Acta.

[27] Holdenrieder S., Nagel D., Schalhorn A., Heinemann V., Wilkowski R., von Pawel J., Raith H., Feldmann K., Kremer A.E., Müller S. (2008). Clinical Relevance of Circulating Nucleosomes in Cancer. Ann. N. Y. Acad. Sci..

[28] Zhu D., Wang H., Wu W., Geng S., Zhong G., Li Y., Guo H., Long G., Ren Q., Luan Y. (2023). Circulating Cell-Free DNA Fragmentation Is a Stepwise and Conserved Process Linked to Apoptosis. BMC Biol..

[29] Wang B.G., Huang H.-Y., Chen Y.-C., Bristow R.E., Kassauei K., Cheng C.-C., Roden R., Sokoll L.J., Chan D.W., Shih I.-M. (2003). Increased Plasma DNA Integrity in Cancer Patients. Cancer Res..

[30] Umetani N., Giuliano A.E., Hiramatsu S.H., Amersi F., Nakagawa T., Martino S., Hoon D.S.B. (2006). Prediction of Breast Tumor Progression by Integrity of Free Circulating DNA in Serum. J. Clin. Oncol..

[31] McInnes M.D.F., Moher D., Thombs B.D., McGrath T.A., Bossuyt P.M., Clifford T., Cohen J.F., Deeks J.J., Gatsonis C., the PRISMA-DTA Group (2018). Preferred Reporting Items for a Systematic Review and Meta-Analysis of Diagnostic Test Accuracy Studies: The PRISMA-DTA Statement. JAMA.

[32] Whiting P.F. (2011). QUADAS-2: A Revised Tool for the Quality Assessment of Diagnostic Accuracy Studies. Ann. Intern. Med..

[33] Zamora J., Abraira V., Muriel A., Khan K., Coomarasamy A. (2006). Meta-DiSc: A Software for Meta-Analysis of Test Accuracy Data. BMC Med. Res. Methodol..

[34] Umetani N., Kim J., Hiramatsu S., Reber H.A., Hines O.J., Bilchik A.J., Hoon D.S.B. (2006). Increased Integrity of Free Circulating DNA in Sera of Patients with Colorectal or Periampullary Cancer: Direct Quantitative PCR for ALU Repeats. Clin. Chem..

[35] Jiang W.-W., Zahurak M., Goldenberg D., Milman Y., Park H.L., Westra W.H., Koch W., Sidransky D., Califano J. (2006). Increased Plasma DNA Integrity Index in Head and Neck Cancer Patients. Int. J. Cancer.

[36] Holdenrieder S., Burges A., Reich O., Spelsberg F.W., Stieber P. (2008). DNA Integrity in Plasma and Serum of Patients with Malignant and Benign Diseases. Ann. N. Y. Acad. Sci..

[37] Ellinger J., Bastian P.J., Ellinger N., Kahl P., Perabo F.G., Buettner R., Mueller S.C., von Ruecker A. (2008). Apoptotic DNA Fragments in Serum of Patients with Muscle Invasive Bladder Cancer: A Prognostic Entity. Cancer Lett..

[38] Gao Y.-J., He Y.-J., Yang Z.-L., Shao H.-Y., Zuo Y., Bai Y., Chen H., Chen X.-C., Qin F.-X., Tan S. (2010). Increased Integrity of Circulating Cell-Free DNA in Plasma of Patients with Acute Leukemia. Clin. Chem. Lab. Med..

[39] Hauser S., Zahalka T., Ellinger J., Fechner G., Heukamp L.C., Von Ruecker A., Müller S.C., Bastian P.J. (2010). Cell-Free Circulating DNA: Diagnostic Value in Patients with Renal Cell Cancer. Anticancer Res..

[40] Pinzani P., Salvianti F., Zaccara S., Massi D., De Giorgi V., Pazzagli M., Orlando C. (2011). Circulating Cell-Free DNA in Plasma of Melanoma Patients: Qualitative and Quantitative Considerations. Clin. Chim. Acta.

[41] Sriram K.B., Relan V., Clarke B.E., Duhig E.E., Windsor M.N., Matar K.S., Naidoo R., Passmore L., McCaul E., Courtney D. (2012). Pleural Fluid Cell-Free DNA Integrity Index to Identify Cytologically Negative Malignant Pleural Effusions Including Mesotheliomas. BMC Cancer.

[42] Hauser S., Kogej M., Fechner G., Von Ruecker A., Bastian P.J., Von Pezold J., Vorreuther R., Lümmen G., Müller S.C., Ellinger J. (2012). Cell-Free Serum DNA in Patients with Bladder Cancer: Results of a Prospective Multicenter Study. Anticancer Res..

[43] Chen H., Sun L.-Y., Zheng H.-Q., Zhang Q.-F., Jin X.-M. (2012). Total Serum DNA and DNA Integrity: Diagnostic Value in Patients with Hepatitis B Virus-Related Hepatocellular Carcinoma. Pathology.

[44] Feng J., Gang F., Li X., Jin T., Houbao H., Yu C., Guorong L. (2013). Plasma Cell-Free DNA and Its DNA Integrity as Biomarker to Distinguish Prostate Cancer from Benign Prostatic Hyperplasia in Patients with Increased Serum Prostate-Specific Antigen. Int. Urol. Nephrol..

[45] Stoetzer O.J., Lehner J., Fersching-Gierlich D., Nagel D., Holdenrieder S. (2014). Diagnostic Relevance of Plasma DNA and DNA Integrity for Breast Cancer. Tumor Biol..

[46] Hao T.B., Shi W., Shen X.J., Qi J., Wu X.H., Wu Y., Tang Y.Y., Ju S.Q. (2014). Circulating Cell-Free DNA in Serum as a Biomarker for Diagnosis and Prognostic Prediction of Colorectal Cancer. Br. J. Cancer.

[47] Leszinski G., Lehner J., Gezer U., Holdenrieder S. (2014). Increased DNA Integrity in Colorectal Cancer. In Vivo.

[48] El-Gayar D., El-Abd N., Hassan N., Ali R. (2016). Increased Free Circulating DNA Integrity Index as a Serum Biomarker in Patients with Colorectal Carcinoma. Asian Pac. J. Cancer Prev..

[49] Kamel A.M., Teama S., Fawzy A., El Deftar M. (2016). Plasma DNA Integrity Index as a Potential Molecular Diagnostic Marker for Breast Cancer. Tumor Biol..

[50] Huang A., Zhang X., Zhou S.-L., Cao Y., Huang X.-W., Fan J., Yang X.-R., Zhou J. (2016). Plasma Circulating Cell-Free DNA Integrity as a Promising Biomarker for Diagnosis and Surveillance in Patients with Hepatocellular Carcinoma. J. Cancer.

[51] Szpechcinski A., Rudzinski P., Kupis W., Langfort R., Orlowski T., Chorostowska-Wynimko J. (2016). Plasma Cell-Free DNA Levels and Integrity in Patients with Chest Radiological Findings: NSCLC versus Benign Lung Nodules. Cancer Lett..

[52] Eltorgoman A.E., Badr E.A.E., Kombr Y.F.A.E., Yousif M. (2017). Pleural Fluid DNA Integrity Index as a Diagnostic Marker of Malignant Pleural Effusion. Br. J. Biomed. Sci..

[53] Leng S., Zheng J., Jin Y., Zhang H., Zhu Y., Wu J., Xu Y., Zhang P. (2018). Plasma Cell-Free DNA Level and Its Integrity as Biomarkers to Distinguish Non-Small Cell Lung Cancer from Tuberculosis. Clin. Chim. Acta.

[54] Kumari S., Husain N., Agarwal A., Neyaz A., Gupta S., Chaturvedi A., Lohani M., Sonkar A.A. (2019). Diagnostic Value of Circulating Free DNA Integrity and Global Methylation Status in Gall Bladder Carcinoma. Pathol. Oncol. Res..

[55] Tang Z., Li L., Shen L., Shen X., Ju S., Cong H. (2018). Diagnostic Value of Serum Concentration and Integrity of Circulating Cell-Free DNA in Breast Cancer: A Comparative Study with CEA and CA15-3. Lab. Med..

[56] Hussein N.A., Mohamed S.N., Ahmed M.A. (2019). Plasma ALU-247, ALU-115, and CfDNA Integrity as Diagnostic and Prognostic Biomarkers for Breast Cancer. Appl. Biochem. Biotechnol..

[57] Zhang R., Pu W., Zhang S., Chen L., Zhu W., Xiao L., Xing C., Li K. (2018). Clinical Value of ALU Concentration and Integrity Index for the Early Diagnosis of Ovarian Cancer: A Retrospective Cohort Trial. PLoS ONE.

[58] Soliman S.E.-S., Alhanafy A.M., Habib M.S.E., Hagag M., Ibrahem R.A.L. (2018). Serum Circulating Cell Free DNA as Potential Diagnostic and Prognostic Biomarker in Non Small Cell Lung Cancer. Biochem. Biophys. Rep..

[59] Qiu Y.W., Shen X.J., Jin C.J., Cao X.J., Ju S.Q. (2018). Value of the Concentration and Integrity of Serum Cell-Free DNA for the Clinical Diagnosis of Esophageal Carcinoma. Zhonghua Zhong Liu Za Zhi.

[60] Yu Z., Qin S., Wang H. (2019). Alter Circulating Cell-Free DNA Variables in Plasma of Ovarian Cancer Patients. J. Obstet. Gynaecol. Res..

[61] Sinha S.K., Brown H., Fang Z., Couetoux M., Gambaro K., Batist G. (2018). A Multiplexed RE-QPCR Cell-Free DNA Assay to Assess Response and Resistance to Cancer Therapy. Cancer Res..

[62] Ren S., Ren X.-D., Guo L.-F., Qu X.-M., Shang M.-Y., Dai X.-T., Huang Q. (2020). Urine Cell-Free DNA as a Promising Biomarker for Early Detection of Non-Small Cell Lung Cancer. J. Clin. Lab. Anal..

[63] Salem R., Ahmed R., Shaheen K., Abdalmegeed M., Hassan H. (2020). DNA Integrity Index as a Potential Molecular Biomarker in Colorectal Cancer. Egypt. J. Med. Hum. Genet..

[64] Park M.-K., Lee J.-C., Lee J.-W., Hwang S.-J. (2021). Alu Cell-Free DNA Concentration, Alu Index, and LINE-1 Hypomethylation as a Cancer Predictor. Clin. Biochem..

[65] Kamal M.M., Abdelaziz A.O., El-Baz H.N., Mohamed G.M., Saleh S.S., Nabeel M.M., Elbaz T.M., Lithy R., Shousha H.I. (2022). Plasma Cell-Free DNA Integrity Index and Hepatocellular Carcinoma Treated or Not with Direct-Acting Antivirals: A Case-Control Study. Arab. J. Gastroenterol..

[66] Sherif F.F., El Desouky M.A., Gebril M., Azmy O.M. (2021). Association of Plasma DNA Integrity and Long Fragment ALU247 in the Diagnosis of Epithelial Ovarian Cancer. Asia Pac. J. Mol. Biol. Biotechnol..

[67] Eldeeb S., Afandy A., Ahmed O., Khodeer S., Younes F., Hosny D. (2021). Plasma Circulating Cell-Free DNA Integrity as a Noninvasive Diagnostic Tool in Hepatocellular Carcinoma. Menoufia Med. J..

[68] Elhelaly R., Effat N., Hegazy M.A.E.-F., Abdelwahab K., Hamdy O., Hashem E.M.A., Elzehery R.R. (2022). Circulating Cell Free DNA and DNA Integrity Index as Discriminating Tools between Breast Cancer and Benign Breast Disease. Asian Pac. J. Cancer Prev..

[69] Rapado-Gonzalez O., Lopez-Cedrun J.L., Lago-Leston R.M., Abalo A., Rubin-Roger G., Salgado-Barreira A., Lopez-Lopez R., Muinelo-Romay L., Suarez-Cunqueiro M.M. (2022). Integrity and Quantity of Salivary Cell-Free DNA as a Potential Molecular Biomarker in Oral Cancer: A Preliminary Study. J. Oral. Pathol. Med..

[70] Kumar S., Nadda N., Paul S., Gamanagatti S., Dash N.R., Vanamail P., Saraya A., Shalimar, Nayak B. (2022). Evaluation of the Cell-Free DNA Integrity Index as a Liquid Biopsy Marker to Differentiate Hepatocellular Carcinoma from Chronic Liver Disease. Front. Mol. Biosci..

[71] Klimaite R., Kazokaite M., Kondrotiene A., Dauksiene D., Verkauskiene R., Zilaitiene B., Dauksa A. (2022). Diagnostic Value of Circulating Cell-Free DNA in Patients with Papillary Thyroid Cancer. Anticancer Res..

[72] Elzehery R., Effat N., El Farahaty R., Elsayed Farag R., Abo-Hashem E.M., Elhelaly R. (2022). Circulating Cell-Free DNA and DNA Integrity as Molecular Diagnostic Tools in Hepatocellular Carcinoma. Am. J. Clin. Pathol..

[73] Mettler E., Fottner C., Bakhshandeh N., Trenkler A., Kuchen R., Weber M.M. (2022). Quantitative Analysis of Plasma Cell-Free DNA and Its DNA Integrity and Hypomethylation Status as Biomarkers for Tumor Burden and Disease Progression in Patients with Metastatic Neuroendocrine Neoplasias. Cancers.

[74] Ren S., Zeng G., Yi Y., Liu L., Tu H., Chai T., Hu L. (2023). Combinations of Plasma CfDNA Concentration, Integrity and Tumor Markers Are Promising Biomarkers for Early Diagnosis of Non-Small Cell Lung Cancer. Heliyon.

[75] Kumari S., Mishra S., Anand N., Hadi R., Rastogi M., Husain N. (2023). Circulating Free DNA Integrity Index and Promoter Methylation of Tumor Suppressor Gene *P16*, *DAPK* and *RASSF1A* as a Biomarker for Oropharyngeal Squamous Cell Carcinoma. Pathol. Res. Pract..

[76] Huang Q., Ji M., Li F., Li Y., Zhou X., Hsueh C., Zhou L. (2023). Diagnostic and Prognostic Value of Plasma Cell-Free DNA Combined with VEGF-C in Laryngeal Squamous Cell Carcinoma. Mol. Cell Probes.

[77] Ren S., Yu C., Huang Q. (2024). Diagnostic Value of Combined Detection of Plasma CfDNA Concentration and Integrity in NSCLC. Lung Cancer Manag..

[78] Siegel R.L., Miller K.D., Fuchs H.E., Jemal A. (2021). Cancer Statistics, 2021. CA Cancer J. Clin..

[79] Hou J., Li X., Xie K.-P. (2021). Coupled Liquid Biopsy and Bioinformatics for Pancreatic Cancer Early Detection and Precision Prognostication. Mol. Cancer.

[80] Zaher E.R., Anwar M.M., Kohail H.M.A., El-Zoghby S.M., Abo-El-Eneen M.S. (2013). Cell-Free DNA Concentration and Integrity as a Screening Tool for Cancer. Indian J. Cancer.

[81] Jiang X., Li H., Liu J., Sun H., Zhang L., Li W., Yao J., Cheng Y. (2020). Feasibility Analysis of Cell-Free DNA Derived from Plasma of Lung Cancer Patients for Next-Generation Sequencing. Biopreserv. Biobank..

[82] Schou J.V., Larsen F.O., Sørensen B.S., Abrantes R., Boysen A.K., Johansen J.S., Jensen B.V., Nielsen D.L., Spindler K.L. (2018). Circulating Cell-Free DNA as Predictor of Treatment Failure after Neoadjuvant Chemo-Radiotherapy before Surgery in Patients with Locally Advanced Rectal Cancer. Ann. Oncol..

[83] Mandel P., Metais P. (1948). Nuclear Acids In Human Blood Plasma. C. R. Seances Soc. Biol. Fil..

[84] Leon S.A., Shapiro B., Sklaroff D.M., Yaros M.J. (1977). Free DNA in the Serum of Cancer Patients and the Effect of Therapy. Cancer Res..

[85] Breitbach S., Tug S., Simon P. (2012). Circulating Cell-Free DNA. Sports Med..

[86] Andreatta M.V., Curty V.M., Coutinho J.V.S., Santos M.Â.A., Vassallo P.F., de Sousa N.F., Barauna V.G. (2018). Cell-Free DNA as an Earlier Predictor of Exercise-Induced Performance Decrement Related to Muscle Damage. Int. J. Sports Physiol. Perform..

[87] Oellerich M., Kanzow P., Walson P.D. (2017). Therapeutic Drug Monitoring - Key to Personalized Pharmacotherapy. Clin. Biochem..

[88] Urosevic N., Merritt A.J., Inglis T.J.J. (2022). Plasma CfDNA Predictors of Established Bacteraemic Infection. Access Microbiol..

[89] Hou Y., Meng X.-Y., Zhou X. (2024). Systematically Evaluating Cell-Free DNA Fragmentation Patterns for Cancer Diagnosis and Enhanced Cancer Detection via Integrating Multiple Fragmentation Patterns. Adv. Sci..

[90] Cristiano S., Leal A., Phallen J., Fiksel J., Adleff V., Bruhm D.C., Jensen S.Ø., Medina J.E., Hruban C., White J.R. (2019). Genome-Wide Cell-Free DNA Fragmentation in Patients with Cancer. Nature.

[91] Leal A.I.C., Mathios D., Jakubowski D., Johansen J.S., Lau A., Wu T., Cristiano S., Medina J.E., Phallen J., Bruhm D.C. (2023). Cell-Free DNA Fragmentomes in the Diagnostic Evaluation of Patients with Symptoms Suggestive of Lung Cancer. Chest.

[92] Jarmuzek P., Wawrzyniak-Gramacka E., Morawin B., Tylutka A., Zembron-Lacny A. (2024). Diagnostic and Prognostic Value of Circulating DNA Fragments in Glioblastoma Multiforme Patients. Int. J. Mol. Sci..

[93] Cheng T.H.T., Jiang P., Tam J.C.W., Sun X., Lee W.-S., Yu S.C.Y., Teoh J.Y.C., Chiu P.K.F., Ng C.-F., Chow K.-M. (2017). Genomewide Bisulfite Sequencing Reveals the Origin and Time-Dependent Fragmentation of Urinary CfDNA. Clin. Biochem..

[94] Nadano D., Yasuda T., Kishi K. (1993). Measurement of Deoxyribonuclease I Activity in Human Tissues and Body Fluids by a Single Radial Enzyme-Diffusion Method. Clin. Chem..

[95] Tsui N.B.Y., Jiang P., Chow K.C.K., Su X., Leung T.Y., Sun H., Chan K.C.A., Chiu R.W.K., Lo Y.M.D. (2012). High Resolution Size Analysis of Fetal DNA in the Urine of Pregnant Women by Paired-End Massively Parallel Sequencing. PLoS ONE.

[96] Burnham P., Dadhania D., Heyang M., Chen F., Westblade L.F., Suthanthiran M., Lee J.R., De Vlaminck I. (2018). Urinary Cell-Free DNA Is a Versatile Analyte for Monitoring Infections of the Urinary Tract. Nat. Commun..

[97] Mouliere F., Chandrananda D., Piskorz A.M., Moore E.K., Morris J., Ahlborn L.B., Mair R., Goranova T., Marass F., Heider K. (2018). Enhanced Detection of Circulating Tumor DNA by Fragment Size Analysis. Sci. Transl. Med..

