# Supplementary material for: Liquid Biopsies Based on Cell-Free DNA Integrity as a Biomarker for Cancer Diagnosis: A Meta-Analysis
Source: Diagnostics (Basel). 2024 Jul 9;14(14):1465. doi: 10.3390/diagnostics14141465 (PMC11276058; doi:10.3390/diagnostics14141465)
Supplement: Supplementary file 1 [file diagnostics-14-01465-s001.zip › diagnostics-3045494-supplementary.pdf]

Supplementary Material

Liquid Biopsies Based on Cell-Free DNA Integrity as a Biomarker for Cancer Diagnosis: A Meta-Analysis

Supplementary Figures

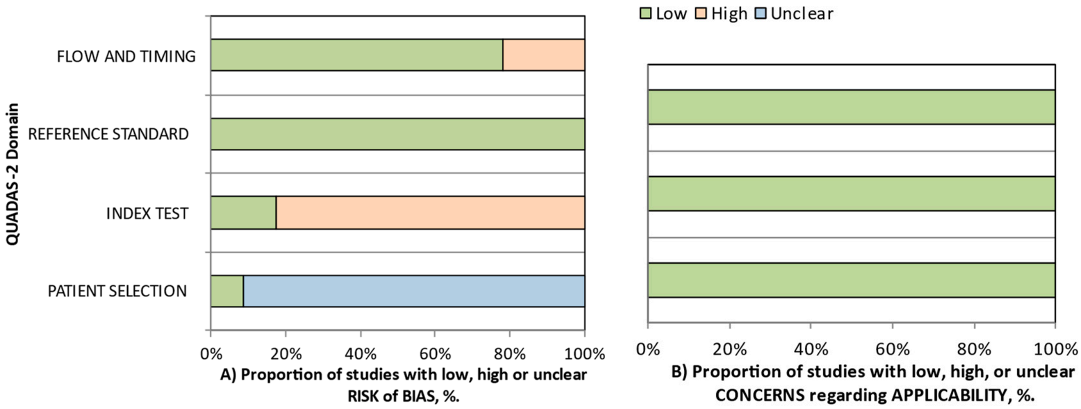

Figure S1. Quality assessment of the included studies according to Quality Assessment of Diagnostic Accuracy Studies-2 (QUADAS-2) template. (A) Proportion of studies with low, high, or unclear concerns regarding applicability. (B) Proportion of studies with low, high, or unclear risk of bias.

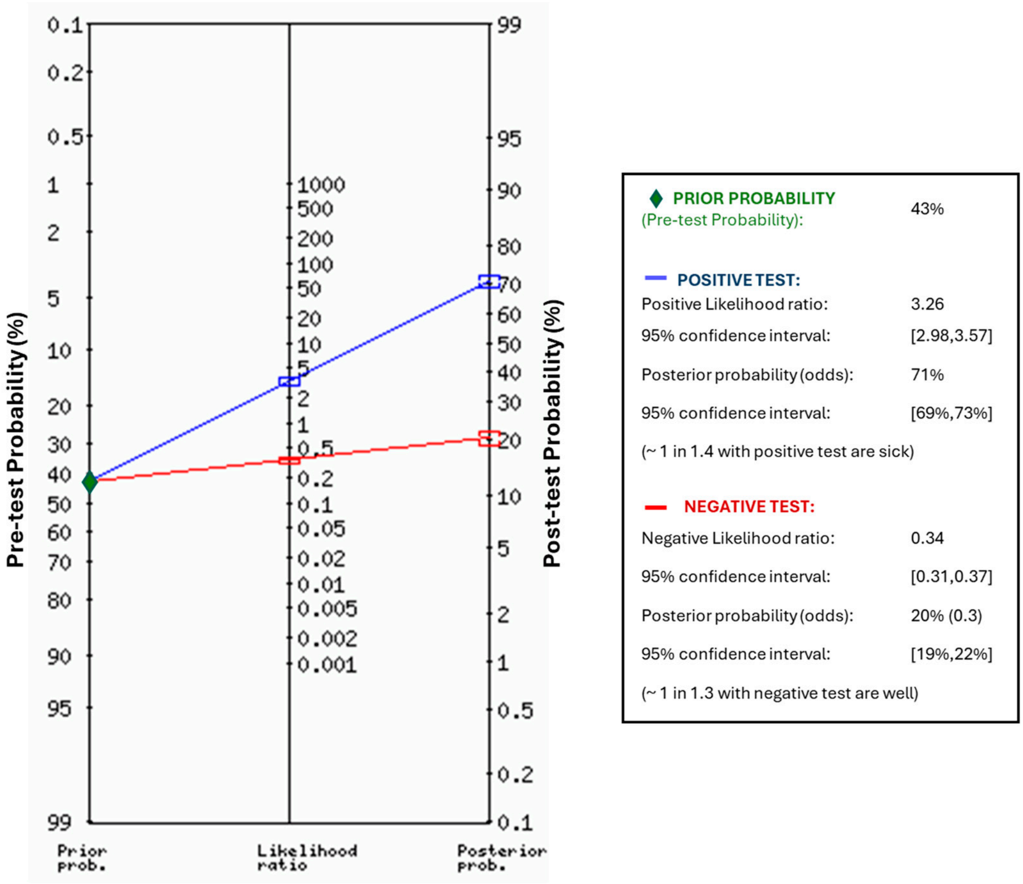

Figure S2. Fagan's monogram evaluating the clinical utility of cfDI in liquid biopsies for differentiating cancer patients. *Abbreviations*: cfDI = cell-free DNA integrity.

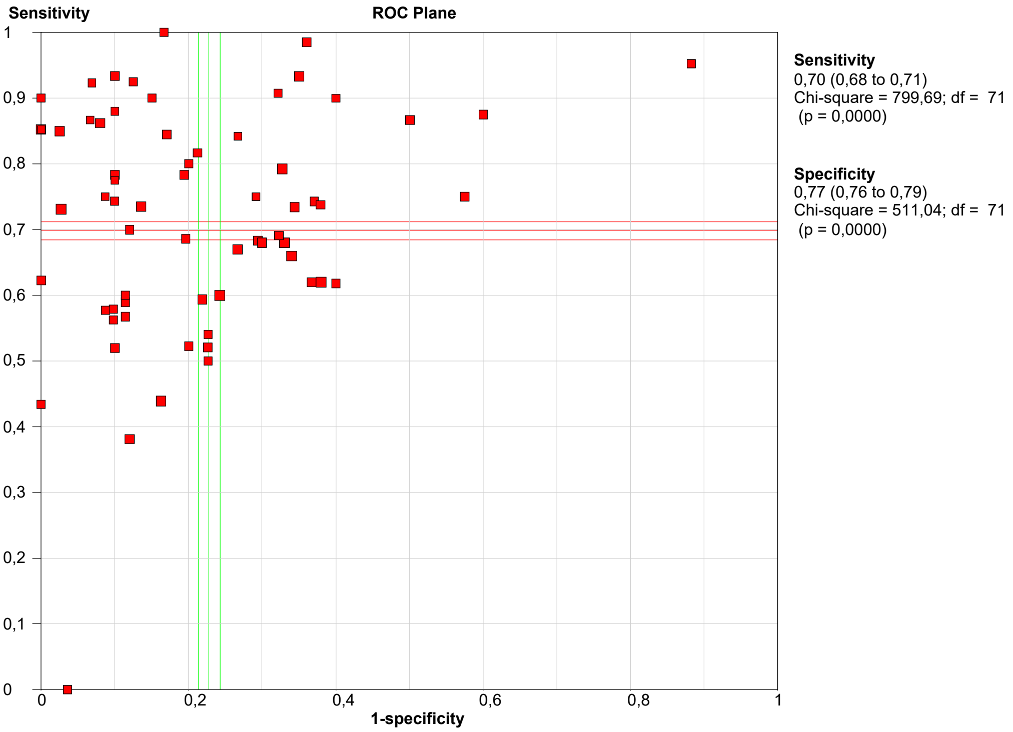

Figure S3. SROC (summary receiver operator characteristic) space for the assessment of the threshold effect of cfDI in liquid biopsies on cancer. *Abbreviations:* ROC = receiver operator characteristic, df = degrees of freedom, cfDI = cell-free DNA Integrity.

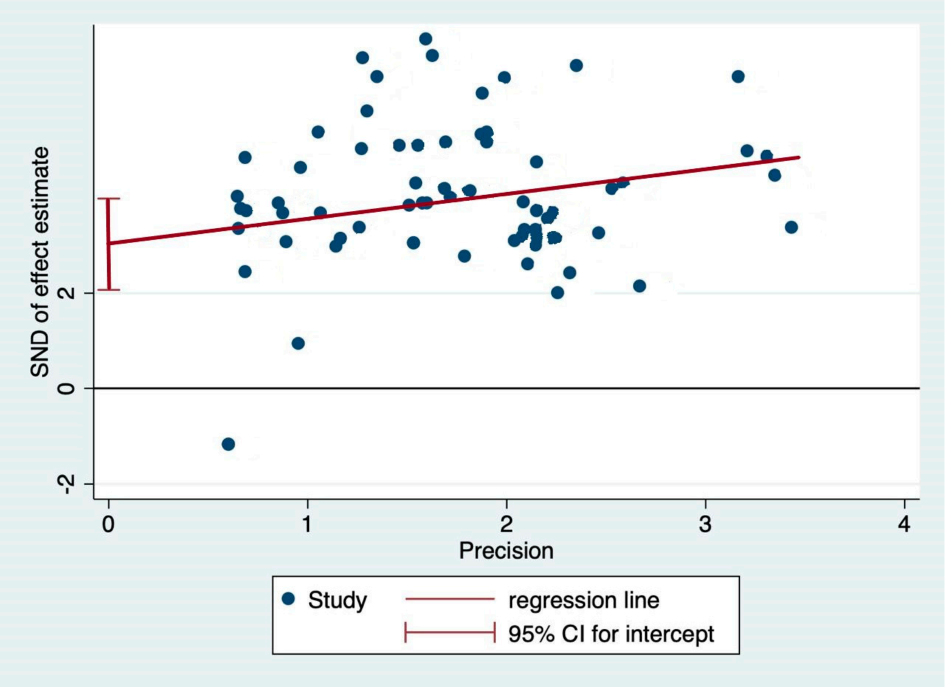

Figure S4. Deeks' funnel plot asymmetry test for the assessment of potential bias of included studies. Each blue dot represents an individual study. The y-axis shows the Standard Normal Deviate (SND) of the effect estimates the x-axis represents the precision of the studies, measured as the inverse of the standard error (1/SE). The red regression line indicates the relationship between the SND of effect estimates and study precision, with the 95% confidence interval (CI) for the intercept depicted as horizontal red lines.

Supplementary Tables

Table S1. Results of regression meta-analysis.

Var.	Coeff.	Std. Err.	*p* - value	RDOR	[95%CI]
Cte.	2.329	0.8188	0.0059	----	----
S	-0.023	0.0872	0.7897	----	----
Sample size	0.182	0.2867	0.5273	1.2	(0.68-2.13)
Type of biofluid	-0.001	0.2351	0.9978	1	(0.62-1.60)
Type of control	0.257	0.1857	0.1703	1.29	(0.89-1.87)
Targeted sequence	-0.457	0.3006	0.1334	0.63	(0.35-1.15)

Tau-squared estimate = 1.0145 (Convergence is achieved after 10 iterations)
Restricted Maximum Likelihood estimation (REML)
No. studies = 72
Filter OFF
Add 1/2 to all cells of the studies with zero

Abbreviations: CI = Confidence Interval; Coeff. = Coefficient; Std. Err. = Standard Error; RDOR = Relative Diagnostic Odds Ratio, Var = Variable.
